# Burden and antimicrobial resistance of *S. aureus* in dairy farms in Mekelle, Northern Ethiopia

**DOI:** 10.1186/s12917-020-2235-8

**Published:** 2020-01-22

**Authors:** Alem Abrha Kalayu, Daniel Asrat Woldetsadik, Yimtubezinash Woldeamanuel, Shu-Hua Wang, Wondwossen A. Gebreyes, Tadesse Teferi

**Affiliations:** 1 Department of Microbiology, Yekatit 12 Hospital Medical College, MCH building, 3rd floor, Room number 321, Addis Ababa, Ethiopia; 2 Department of Microbiology, Immunology and Parasitology; College of Health Sciences, Addis Ababa University, Addis Ababa, Ethiopia; 3 Infectious Disease Division, Internal Medicine Department, College of Medicine, the Ohio State University, Columbus, USA; 4 Molecular Epidemiology, College of Veterinary Medicine, the Ohio State University, Columbus, USA; 5 Tropical Veterinary Medicine, College of Veterinary Medicine, Mekelle University, Mekelle, Ethiopia

**Keywords:** *Staphylococcus aureus*, MRSA, Antimicrobial resistance, Dairy cows, Mekelle

## Abstract

**Background:**

*Staphylococcus aureus* is a frequent colonizer of human and several animal species, including dairy cows. It is the most common cause of intramammary infections in dairy cows. Its public health importance increases inline to the continuous emergence of drug-resistant strains; such as Methicillin-resistant *S. aureus* (MRSA). Indeed, the recent emergence of human and veterinary adapted MRSA demands serious attention. The aim of this study was to determine the burden and drug resistance pattern of *S. aureus* in dairy farms in Mekelle and determine the molecular characteristics of MRSA.

**Results:**

This study was done on 385 lactating dairy cows and 71 dairy farmers. The ages of the cows and farmworkers were between 3 and 14 and 17–63 years respectively. *S. aureus* was isolated from 12.5% of cows and 31% of farmworkers. Highest resistance was observed for penicillin (> 90%) followed by tetracycline (32–35%) and trimethoprim-sulphamethoxazole (10–27%). But no resistance was observed for vancomycin, daptomycin, and rifampin. Only one isolate was MRSA both phenotypically and harboring *mecA*. This isolate was from nasal of a farmworker and was MRSA SCC*mec* Iva, *spa* type t064 of CC8. Multi-drug resistance was observed in 6.2% of cow isolates and 13.6% of nasal isolates.

**Conclusions:**

In this study, *S. aureus* infected 12.5% of dairy cows and colonized 31% of farmworkers. Except for penicillin, resistance to other drugs was rare. Although no MRSA was found from dairy cows the existence of the human and animal adapted and globally spread strain, MRSA SCC*mec* IVa spa t064, warrants for a coordinated action to tackle AMR in both human and veterinary in the country.

## Background

*Staphylococcus aureus* causes a diverse array of diseases in both human and animals, including dairy cows. Human infections range from mild skin infections to life-threatening ones; such as bacteremia, endocarditis, necrotizing pneumonia and toxic shock syndrome (TSS) [1]. The major diseases in animals include mastitis (infection of the intramammary gland) in dairy cows, joint infections in poultry and surgical site infections equine [2]. Intramammary infections of dairy cows negatively affect the dairy industry due to poor milk yields, veterinary treatments, and milk that must be discarded [3]. Besides causing diseases, *S. aureus* commonly colonizes and lives harmlessly in different body parts of human and animals. About half of healthy human individuals are thought to be colonized persistently or transiently [2] with the most common sites being the anterior nares [4]. The teat skin, rectum or nasal cavity of dairy cows are also commonly colonized by *S. aureus* [3]. *S. aureus* transmission in dairy cattle is thought to occur primarily via milking machine, udder cloths or milkers’ hands [5].

*S. aureus* is a highly adaptive pathogen which continuously evolves resistance to most of the available antibiotics [6]. The best example is the emergence and spread of MRSA initially in the healthcare setting later in the community among relatively young and healthy individuals [7] and recently in livestock and people having occupational contact with these animals [4]. Factors leading to drug resistance in *S. aureus* include acquisition of mobile genetic elements (MGEs) carrying resistance genes [8] and improper use of drugs. Antimicrobial use in food-producing animals for growth promotion and/or treatment purpose is facilitating the selection and spread of resistant bacteria in these animals. Such bacteria could be transmitted to humans via food and other transmission routes [9, 10].

Methicillin resistance is caused by the acquisition of the *mecA* or *mecC* gene in the mobile genetic element called SCC*mec*. Both of the *mec* genes code for alternative penicillin-binding proteins, PBP2a, that has reduced affinity to most β-lactam antibiotics [11]. Traditionally, most MRSA strains are known to have specific host tropism. However, recent reports showed that some strains are losing their specificity and be easily transmitted from animals to humans or vice-versa [12]. For example, the MRSA of ST1-SCC*mec* IV which have been known to be associated with community-acquired infections in a human was isolated from dairy cows with mastitis in Australia [13] and Italy [14]. In addition, the epidemic human strain, UK-EMRSA-15 of CC22, was demonstrated recently in intramammary infections of dairy cows in Italy [12]. Livestock acquired (LA) MRSA due to *mecC*, which has spread extensively in livestock animals, has emerged recently among high-risk groups who have occupational contact with these animals [4].

In Ethiopia, there is strong research-based evidence showing *S. aureus* as an important udder pathogen in dairy cows [15–18]. However, whether *mecA/mecC* harbouring *S. aureus* strains are associated with dairy cows infection is not well explored. So far, two studies by Tigabu et al. [19] and Mekonnen et al. [20] attempted to detect *mecA* positive strains from dairy milk but neither found it. However, it should be noted that both studies targeted only *mecA* but not *mecC* (another gene responsible for methicillin resistance). As to our knowledge, study to detect MRSA from dairy cows and farmworkers to demonstrate the existence of adapted strains hasn’t been conducted so far in Ethiopia.

Therefore, the present study aimed to determine the burden of *S. aureus* and its drug resistance pattern in dairy cows and farmworkers and study the molecular characteristics of MRSA.

## Results

In this study a total of 385 lactating dairy cows and 71 dairy farm workers from 67 dairy farms located in 3 sub-cities of Mekelle, Northern Ethiopia were studied*.*

The cows’ age ranged from 3 to 14 years with a mean age of 5.9 years. Majority of them were within the age group of 3–5 years (178/385, 46.2%). The parity of the cows ranged from 1 to 11 where two-third (254/385, 66%) of them gave birth to 1–3 calves. More than half of the cows (203/385, 52.7%) had less than 3 months of lactation stage. In all the dairy farms the milking process was done by manual method (Table 1).

**Table 1:** Characteristics of dairy cows from 67 small and large scale dairy farms in Mekelle, Northern Ethiopia

Variables	Sub-city and number of lactating cows			Total, n/385 (%)
	Hadnet (*n* = 84) (%)	Hawolti (*n* = 200) (%)	Semen (*n* = 101) (%)	
Age group in years				
3–5 years	39 (46.4)	94 (47.0)	45 (44.6)	178 (46.2)
6–8 years	34 (40.5)	87 (43.5)	47 (46.5)	168 (43.6)
≥ 9 years	11 (13.1)	19 (9.5)	9 (8.9)	39 (10.1)
Parity				
1–3	52 (61.9)	135 (67.5)	67 (66.3)	254 (66.0)
4–6	28 (33.3)	53 (26.5)	32 (31.7)	113 (29.4)
> 6	4 (4.8)	12 (6.0)	2 (2.0)	18 (4.7)
Lactation				
< 3 month	59 (70.2)	90 (45.0)	54 (53.5)	203 (52.7)
3–6 month	25 (29.8)	63 (31.5)	39 (38.6)	127 (33.0)
> 6 month	0 (0)	47 (23.5)	8 (7.9)	55 (14.3)

Regarding the dairy farmworkers, more than three fourth (55/71, 77.5%) were males and their age ranged from 17 to 63 years with the mean age of 29 years. They had a work experience of up to 40 years in the current farm (Table 2).

**Table 2:** Sociodemographic characteristics of dairy farmworkers from 67 small and large scale dairy farms in Mekelle, Northern Ethiopia

Variables	Frequency (n)	Percent (n/71*100)
Sex		
Male	55	77.5
Female	16	22.5
Age in years, *n* = 71		
17–27	45	63.4
28–38	10	14.1
39–49	7	9.9
50–63	9	12.7
Farm Duty, *n* = 71		
Attendant	62	87.3
Owner	9	12.7
Work experience in the current dairy farm, *n* = 71		
≤ 5 years	50	70.4
6–10 years	14	19.7
11–15 years	1	1.4
16–20 years	4	5.6
≥ 21 years	2	2.8

### The isolation rate of *Staphylococcus aureus*

*S. aureus* was initially identified by biochemical methods and confirmed by *nuc* gene detection. Accordingly, 12.5% (48/385) of udder quarters of dairy cows and 31% (22/71) of nares of dairy farm workers harbored *S. aureus*.

### Antimicrobial susceptibility of isolates

Milk isolates showed the highest resistance for penicillin (44/48, 91.7%) followed by tetracycline (17/48, 35.4%) and trimethoprim-Sulphamethoxazole (5/48, 10.4%). No MRSA was isolated from the milk of the dairy cows (Table 3). Similarly, nasal isolates also showed the highest resistance for penicillin (20/22, 90.9%) followed by tetracycline (7/22, 31.8%) and trimethoprim-sulphamethoxazole (6/22, 27.3%). One MRSA isolate was documented on a dairy farmer as determined phenotypically by Cefoxitin disk diffusion method. Phenotypically MRSA isolate was not found from the dairy cows. In addition, no resistance was observed for vancomycin, daptomycin, and rifampin in both the milk and nasal isolates (Table 3).

**Table 3:** Antimicrobial susceptibility profile of 193 *S. aureus* isolates from human and dairy cows in Mekelle, Northern Ethiopia

Antibiotic & concentration	Interpretation	Dairy farmers	Dairy cows
Penicillin (10 μg)	S	2 (9.1)	4 (8.3)
	R	20 (90.9)	44 (91.7)
Cefoxitin (30 μg)	S	21 (95.5)	48 (100)
	R	1 (4.5)	0 (0)
Erythromycin (15 μg)	S	18 (81.8)	44 (91.7)
	I	0 (0)	3 (6.2)
	R	4 (18.2)	1 (2.1)
Clindamycin (2 μg)	S	22 (100)	47 (97.9)
	I	0 (0)	0 (0)
	R	0 (0)	1 (2.1)
Tetracycline (30 μg)	S	15 (68.2)	28 (58.3)
	I	0 (0)	3 (6.2)
	R	7 (31.8)	17 (35.4)
Trimethoprim-Sulfamethoxazole (1.25/23.75 μg)	S	16 (72.7)	41 (85.4)
	I	0 (0)	2 (4.2)
	R	6 (27.3)	5 (10.4)
Rifampin (5 μg)	S	22 (100)	48 (100)
Vancomycin E-test	S	22 (100)	48 (100)
Daptomycin E-test	S	22 (100)	48 (100)

Key: *S* Sensitive, *I* Intermediate, *R* Resistant

Overall, 46/48 (95.8%) of the milk isolates were resistant to at least one of the nine antimicrobial agents where 29/48(60.4%) were resistant to only one, 14/48 (29.2%) to two and 3/48 (6.2%) were multi-drug resistant (resistant to three or more drugs in different classes). Regarding the nasal isolates, 20/22 (90.9%) were resistant to at least one, 7/22 (31.8%) to only one, 10/22 (45.5%) to two and 3/22 (13.6%) were MDR.

### Molecular characterization of isolates

All the 70 isolates from cows’ udder quartes (48 isolates) and farmers’ nares (22 isolates) were tested for their *mecA* and *mecC* possession. Only one isolate from the nasal of a farmworker was found positive for *mecA*. No *mecA* was documented from the cows. In addition, All the human and cow isolates were negative for *mecC*. Further characterization of the *mecA* positive *S. aureus* isolates revealed that it was *spa* type t064 and SCC*mec* type Iva. Based on the *spa* sequence this was in the clonal complex 8 (CC8).

## Discussion

*Staphylococcus aureus* commonly colonizes various body parts of human and dairy cows that can lead to a diverse array of diseases [2]. Intramammary infection in dairy cows is common resulting in significant economic loss to farmers due to decreased milk production or abnormal milk that must be discarded [3]. Furthermore, consumption of milk obtained from infected dairy cows by a human can lead to infections or intoxications by toxins produced by *S. aureus* [21, 22]. The emergence of drug-resistant strains, such as MRSA, in both animals and human with the ability of cross-transmission between them, is of special concern [4]. *S. aureus* human and animal cross-transmission is well demonstrated between dairy cattle and farm workers due to their close contact [23]. Hands of such farmers appear to be the primary transmitters of *S. aureus* to the dairy cows during milking [5]. As to our knowledge, there is only one research report on *S. aureus* from both dairy cows and farmers in Ethiopia [24] unlike the several studies conducted on dairy cows. Indeed, this study was limited to phenotypic characterization only. Hence, the present study was conducted on 71 farm workers and 385 dairy cows obtained from 67 dairy farms located in Mekelle, Northern Ethiopia to determine the burden and resistance of *S. aureus* and consequently study the molecular characteristics of MRSA.

In the present study, 31% of dairy farmers were found colonized by *S. aureus* in their nares which is higher than a previous study done around Addis Ababa, Ethiopia where 13.2% of dairy farmers were found colonized [24]. In the present study, tryptic soy broth as an enrichment media was used to maximize the recovery of *S. aureus* from nasal swabs and this might explain the higher frequency of isolation as compared to the similar study conducted in Ethiopia [24]. Studies from other parts of the world also showed a significant nasal carriage of *S. aureus* from farmworkers; 15.2% of dairy farm workers carried *S. aureus* in their nares in South Africa [25] and 36% in Catania, Italy [26].

In the present study, milk samples from 12.5% of dairy cows yielded *S. aureus*. Similarly, previous studies in Ethiopia also reported an *S. aureus* prevalence of 9 to 27.9% [24, 27–29]. Other studies in Western Zambia [30], Zimbabwe [31] and Northern Italy [32] have reported an *S. aureus* isolation rate of 22, 16.3, and 9.1%, respectively from cows’ milk.

The continuous evolution of resistance to most of the available antibiotics by *S. aureus* is a key public health concern. The first antibiotic-resistant *S. aureus* was reported for penicillin 2 years after the introduction of the drug [33]. Since then, penicillin-resistant *S. aureus* has increased and spread widely. Nowadays the majority of *S. aureus* isolates elsewhere are penicillin-resistant [34]. Previous studies in Ethiopia reported penicillin resistance in 97–100% of *S. aureus* isolates from dairy cows and farm workers [24, 29]. In agreement, the present study documented penicillin resistance in 91.7 and 90.9% of cow and farmer isolates respectively. However, resistance to other antimicrobials was found either very low or absent in the current study. The respective resistances of dairy cows and farmworkers’ isolates were tetracycline 35.4% and 31.8, trimethoprim-sulphamethoxazole 10.4 and 27.3% and methicillin 0 and 4.5%. Furthermore, all the isolates were susceptible to vancomycin, daptomycin, and rifampin.

Despite several reports on MRSA from dairy cows elsewhere, the present study didn’t find any isolate harboring *mecA/mecC*. This is in agreement with the previous studies conducted in central Ethiopia [19], Tunisia [35] and Australia [36]. However, up to 52% MRSA was reported from the milk of dairy cows in Egypt [37, 38], 17% in Turkey [39], 11% in Brazil [40], 9.3% in Belgium [41], 6.2% in Korea [42]. From the present and previous studies in Ethiopia, it is possible that MRSA adapted to the intramammary gland of dairy cows is not circulating. However, the present study recommends the conduct of a nationwide study for a better conclusion. Indeed, Ethiopian has to learn from the other countries with high MRSA burden and implement antimicrobial stewardship program in both human and animals.

Unlike dairy cows, MRSA harboring *mecA* was found in one (4.5%) of *S. aureus* isolates from the nares of a dairy farmer. As to our knowledge, this is the first report on *mecA* positive *S. aureus* in Ethiopia. Further characterization showed that the strain was MRSA-SCC*mec* Iva, *spa* type t064 and in the CC8. This MRSA strain was previously reported from human patients with bacteremia in South Africa [43], nasal and blood of human patients in the USA [44], from a veterinary surgeon from South America [45], goats in the Czech Republic [46], human clinical isolates, horses and veterinary personnel in Ireland [47], horse infections in Germany [48], the Netherlands [49] and Sweden [50]. This highlights MRSA *spa* t064 harboring SCC*mec* IVa is an adapted strain to both human and animals with global distribution. As indicated above, this strain is commonly isolated from horse infections and colonization. Hence, future studies should be done to determine its existence in horses in Ethiopia. All the *S. aureus* isolates will be genotyped in the future to see if there are some strains shared by the dairy cows and farmers.

## Conclusions

In the present study, *S. aureus* was detected in the udders of 12.5% of dairy cows and nares of 31% of farmworkers. The isolates from both cows and farmworkers showed high resistance to Penicillin (> 90%), low resistance to tetracycline and trimethoprim-sulphamethoxazole but no resistance for vancomycin, daptomycin, and rifampin. This study reported MRSA SCC*mec* IVa, spa type t064 for the first time in the country. This strain was previously reported from human and veterinary in different parts of the continent. This warrants a coordinated one health approach to contain and prevent the emergence and spread of such drug-resistant microbes in Ethiopia. As it was commonly reported from horses, conducts of future studies to determine whether such animals harbor this strain are strongly recommended in Ethiopia.

## Methods

### Study area

This study was carried out in Mekelle, the capital city of the Tigray Regional State, Northern Ethiopia. According to the 2017 report by the central statistical agency of Ethiopia, the cattle population of the region was around 4.8 million where around 2.4 million (51%) were females; among these 28,133 were dairy cows [51].

### Study design and period

A cross-sectional study was conducted from March 2016 to March 2017 to characterize *S. aureus* isolates from dairy cows and farmworkers.

### Study population

This study was done on 385 lactating dairy cows and 71 dairy farmers from 67 Small and large scale dairy farms located in 3 sub-cities of the town; i.e. Hawolti, Semen, and Hadnet. All lactating cows and all available dairy farmworkers during data/sample collection were included.

### Data and sample collection

Data regarding sociodemographic characteristics of the dairy cows and farmers were collected using questioner. Also, appropriate laboratory samples from all the study participants were collected.

#### A nasal swab from dairy farmers

Swabs were collected from both nares of dairy farm workers using BD culture swab (Becton, Dickinson and Company, USA). The sterile swab was inserted about 2.5 cm (1 in.) from the edge of the nares and rotated 5 times against the anterior nasal mucosa and repeated with the same swab in the second naris. The nasal swab was then returned to its tube, labeled and transported to the Microbiology laboratory of Ayder Referal Hospital in Mekelle, Northern Ethiopia.

#### Milk from dairy cows

Pooled milk sample was collected from all udder quarters of each lactating dairy cow according to the procedures of the National Mastitis Council. Briefly, the udders of the cow were thoroughly cleaned with water and dried with a clean towel. Then teat ends were disinfected with cotton swabs soaked in 70% alcohol and allowed to air dry. After discarding the first streams; three to four streams of milk (1–2 ml) from each udder quarter (4 × 3 = 12 streams in total from a single cow) were collected into a sterile leak-proof plastic container and transported to the Microbiology laboratory in an icebox.

### Culture and identification of *S. aureus*

All specimens were processed for culture and sensitivity testing. Nasal swabs were initially incubated overnight in Brain Heart Infusion (BHI) broth (Oxoid, Ltd., England) to increase the recovery of *S. aureus*. Then 10 μl of milk and 100 μl of the broth were each inoculated into blood agar containing 5% sheep blood (Oxoid, Ltd., England) and Mannitol salt agar (Oxoid). The plates were incubated at 35–37 °C in an anaerobic atmosphere for 24 h and then inspected for bacterial growth. Incubation was extended to 48 h if no growth was observed within 24 h. Colonies suspected as *S. aureus* were sub-cultured into Tryptic soy agar to get pure colonies and were identified as *S. aureus* based on colony characteristics, Gram stain reaction, hemolysis, catalase test, coagulase test, DNase test, and mannitol fermentation. Phenotypically identified *S. aureus* isolates were stored in 20% glycerol at − 70 °C until they were shipped to the Ohio State University, the USA for molecular characterization.

### Antimicrobial susceptibility testing

Antimicrobial susceptibility testing (AST) was done using disc diffusion technique and E-test according to the criteria of the Clinical and Laboratory Standards Institute [52]. Briefly, a standardized suspension of each *S. aureus* isolate was prepared using normal saline standardized using McFarland 0.5. The standardized suspension was streaked on to Muller-Hinton Agar (Oxoid) and allowed to dry. Then, the antibiotic discs or E-test strips were placed on the medium and incubated at 35–37 °C for 16–18 h. The incubation time was extended to 24 h for cefoxitin (30 μg) disc, which was used as a surrogate test for methicillin resistance. After the appropriate incubation time, the zones of inhibition were measured using a caliper and interpreted as sensitive, intermediate and resistant. For the disc diffusion method, the following antimicrobials and disc potencies were used: penicillin (10 IU), cefoxitin (30 μg), erythromycin (15 μg), tetracycline (30 μg), clindamycin (2 μg), trimethoprim-sulphamethoxazole (1.25/23.75 μg) and rifampin (5 μg). However, susceptibility testing for vancomycin and daptomycin was done using the E-test method. Inducible clindamycin resistance was also determined by double-disk diffusion test (D-test) for erythromycin-resistant but clindamycin susceptible isolates.

### DNA extraction

Genomic DNA was extracted using DNeasy Blood and Tissue extraction kit for gram-positive bacteria (Qiagen, Valencia, CA) following the manufacturer’s instructions.

### Polymerase chain reaction (PCR)

PCR was used to confirm the phenotypically identified isolates, detect *mecA/mecC* and SCC*mec* typing at the Infectious Diseases Molecular Epidemiology Laboratory (IDMEL), Department of Veterinary Preventive Medicine, College of Veterinary Medicine, The Ohio State University, USA. The primer pairs and control strains used are shown in Table 4.

**Table 4:** Control strains and primer pairs for the PCRs to detect *nuc, mecA, mecC* genes of *S. aureus* isolated from humans and animals in Mekelle, Ethiopia

Control strains			
Strain	Target genes possessed		
ATCC 29213	*nuc*		
ATCC43300	*mecA*		
LGA251	*mecC*		
Primers pairs			
Genes	Nucleotide sequence (5′ → 3′)	Amplified product size (bp)	Refe.
*nucF*	GCGATTGATGGTGATACGGTT	267	[53]
*nucR*	AGCCAAGCCTTGACGAACTAAAGC		
*mecA P4*	TCCAGATTACAACTTCACCAGG	162	[54]
*mecA P7*	CCACTTCATATCTTGTAACG		
*mecA*_*LGA251*_ MultiFP	GAAAAAAAGGCTTAGAACGCCTC	138	
*mecA*_*LGA251*_ MultiRP	GAAGATCTTTTCCGTTTTCAGC		

***nuc*** **detection:** Phenotypically identified *S. aureus* isolates were confirmed by the detection of the thermonuclear coding gene, *nuc* according to Brakstad et al. [53] using the illustra PuReTaq Ready-To-Go PCR Beads (GE Healthcare Bio-Sciences, USA).

***mecA*,** ***mecC*** **detection:** performed as previously described protocol by Stegger et al. [54] using the illustra PuReTaq Ready-To-Go PCR Beads (GE Healthcare Bio-Sciences, USA).

**SCC*mec*** **typing:** SCC*mec* typing was done according to previously described multiplex PCR by Kondo et al. [55]. All PCR products were analyzed using agarose gel electrophoresis and transilluminator for visualization of bands.

***spa*** **typing:** was performed for all confirmed *S. aureus* isolates as previously described protocol [56] at the Public Health Research Institute, International Center for Public Health, The State University of New Jersey, USA. Shortly, the polymorphic X region of *the spa* gene was amplified by PCR and sequenced. Sequences were analyzed using Ridom Staph-Type software (Ridom GmbH) which detects *spa* repeats automatically and assigns a *spa*-type (http://spaserver.ridom.de/).

### Data analysis

Data was entered into excel spreadsheet, cleaned and exported to SPSS software version 20 for analysis. *P-value < 0.05* was considered as cut off point for the significant association.

### Ethical consideration

This study was conducted after approved by the Institutional Review Board (AAU-IRB) of the Colleges of Health Sciences, Addis Ababa University, Ethiopia and National Research Ethics Review Committee (NERC) of Ministry of Science and Technology, Ethiopia. Also, permission from dairy farm owners/managers was obtained before collection of milk samples. Written informed consent was obtained from each dairy farmer. The aim of the study, its significance, confidentiality, participation right, procedure, and associated risks were explained through an information sheet.

## Data Availability

The datasets used and/or analyzed during the current study are available from the corresponding author on reasonable request.

## Abbreviations

AMR: Antimicrobial resistance
AST: Antimicrobial susceptibility testing
ATCC: American type culture collection
CC: Clonal complex
CLSI: Clinical and Laboratory Standards Institute
MDR: Multi-drug resistance
MRSA: Methicillin-resistant *Staphylococcus aureus*
PBP: Penicillin binding protein
PCR: Polymarase chain reaction
SCC*mec*: Staphylococcal cassette chromosomes *mec*
*spa*: Staphylococcal protein A
ST: Sequence type
